# Disparate and shared transcriptomic signatures associated with cortical atrophy in genetic behavioral variant frontotemporal degeneration

**DOI:** 10.1186/s13024-025-00806-3

**Published:** 2025-02-07

**Authors:** Ting Shen, Jacob W. Vogel, Vivianna M. Van Deerlin, EunRan Suh, Laynie Dratch, Jeffrey S. Phillips, Lauren Massimo, Edward B. Lee, David J. Irwin, Corey T. McMillan

**Affiliations:** 1 Penn Frontotemporal Degeneration Center, Department of Neurology, Perelman School of Medicine, University of Pennsylvania, 3700 Hamilton Walk, Richards 606B, Philadelphia, PA 19104 USA; 2 Department of Clinical Sciences Malmö, SciLifeLab, Lund University, Lund, Sweden; 3 Center for Neurodegenerative Disease Research, Department of Pathology and Laboratory Medicine, Perelman School of Medicine, University of Pennsylvania, Philadelphia, PA USA

**Keywords:** Behavioral variant frontotemporal dementia, Transcriptomics, Cortical thickness, Partial least squares regression, Synaptic density, Pathology

## Abstract

**Background:**

Cortical atrophy is a common manifestation in behavioral variant frontotemporal degeneration (bvFTD), exhibiting spatial heterogeneity across various genetic subgroups, which may be driven by distinct biological mechanisms.

**Methods:**

We employed an integrative imaging transcriptomics approach to identify both disparate and shared transcriptomic signatures associated with cortical thickness in bvFTD with *C9orf72* repeat expansions or pathogenic variants in *GRN* or *MAPT*. Functional enrichment analyses were conducted on each gene list significantly associated with cortical thickness. Additionally, we mapped neurotransmitter receptor/transporter density maps to the cortical thickness maps, to uncover different correlation patterns for each genetic form. Furthermore, we examined whether the identified genes were enriched for pathology-related genes by using previously identified genes linked to TDP-43 positive neurons and genes associated with tau pathology.

**Results:**

For each genetic form of bvFTD, we identified cortical thickness signatures and gene sets associated with them. The cortical thickness associated genes for *GRN*-bvFTD were significantly involved in neurotransmitter system and circadian entrainment. The different patterns of spatial correlations between synaptic density and cortical thinning, further confirmed the critical role of neurotransmission and synaptic signaling in shaping brain structure, especially in the *GRN*-bvFTD group. Furthermore, we observed significant overlap between genes linked to TDP-43 pathology and the gene sets associated with cortical thickness in *C9orf72*-bvFTD and *GRN*-bvFTD but not the *MAPT*-bvFTD group providing specificity for our associations. *C9orf72*-bvFTD and *GRN*-bvFTD also shared genes displaying consistent directionality, with those exhibiting either positive or negative correlations with cortical thickness in *C9orf72*-bvFTD showing the same direction (positive or negative) in *GRN*-bvFTD. *MAPT*-bvFTD displayed more pronounced differences in transcriptomic signatures compared to the other two genetic forms. The genes that exhibited significantly positive or negative correlations with cortical thickness in *MAPT*-bvFTD showed opposing directionality in *C9orf72*-bvFTD and *GRN*-bvFTD.

**Conclusions:**

Overall, this integrative transcriptomic approach identified several new shared and disparate genes associated with regional vulnerability with increased biological interpretation including overlap with synaptic density maps and pathologically-specific gene expression. These findings illuminated the intricate molecular underpinnings contributing to the heterogeneous nature of disease distribution in bvFTD with distinct genetic backgrounds.

**Supplementary Information:**

The online version contains supplementary material available at 10.1186/s13024-025-00806-3.

## Introduction

Behavioral variant frontotemporal dementia (bvFTD) is characterized by a progressive deterioration of personality and social behavior, and often accompanied by cognitive impairments [1]. While the majority of individuals with bvFTD have apparently sporadically illness, a subset of bvFTD have pathogenic variants in genes associated with autosomal dominant inheritance of FTD, with a greater likelihood of a genetic etiology in the presence of family history of FTD or related conditions. Approximately 15–20% individuals with bvFTD have an identifiable genetic etiology [2], with the most prevalent genetic causes including repeat expansions of *C9orf72*, pathogenic variants in *GRN* and *MAPT* genes [3]. Genetic forms are often linked to distinct clinical presentations, underlying pathologies, and neuroanatomical patterns in comparison to apparently sporadic bvFTD, which complicate the disease landscape.

Cortical degeneration is a common neuroanatomical manifestation in bvFTD, primarily affecting the frontal and anterior temporal lobe. Distinct brain atrophy patterns have been observed among different genetic forms. BvFTD with repeat expansions in *C9orf72* (*C9orf72*-bvFTD) exhibits a higher degree of atrophy in parietal and occipital lobes, as well as the lateral inferior frontal lobe and cerebellum, compared with bvFTD carrying *MAPT* pathogenic variants (*MAPT*-bvFTD) and apparently sporadic bvFTD [4, 5]. Compared to *C9orf72*-bvFTD, *MAPT*-bvFTD demonstrates more pronounced atrophy in the anterior temporal lobes, while bvFTD carrying *GRN* pathogenic variants (*GRN*-bvFTD) shows greater grey matter loss in inferior temporal and parietal lobes [4, 5]. Moreover, frontotemporal lobar degeneration (FTLD) with *GRN* pathogenic variants experience faster progression of atrophy than *C9orf72*-FTLD and *MAPT*-FTLD [6]. Distinct atrophy patterns across various forms of genetic bvFTD may be shaped by their different biological mechanisms.

Different genetic forms of bvFTD may exhibit distinct and shared molecular signatures that influence the disease presentation and progression. Genetic variants may lead to dysregulated molecular pathways related to the mutated genes. For instance, the dysfunction of C9orf72 protein may interrupt the regulation of endosomal trafficking, autophagy, lysosomal and microglial functions, and lead to neurodegeneration [7]. The *MAPT* pathogenic variants give rise to a cascade of events that cause the dysregulation of synaptic, neuronal, and lysosomal functions [8]. Within the frontal cortex of *GRN*-FTD, there are upregulated genes associated with TGF-beta signaling and cell communication, while downregulated genes are involved in calcium signaling [9]. Additionally, another study further analyzed transcriptomics data in cerebellum, frontal cortex, and hippocampus, which revealed that dysregulated genes in *GRN*-FTD primarily centered on Wnt signaling pathway [10]. We hypothesize that the regional distribution of gene expression may contribute to selective vulnerability of regional neurodegeneration, giving rise to a variety of distinct neuroanatomic patterns observed in genetic bvFTD. Therefore, it is crucial to consider each genetic form of bvFTD individually to better understand the complex genetic underpinnings of bvFTD.

**Fig. 1:**
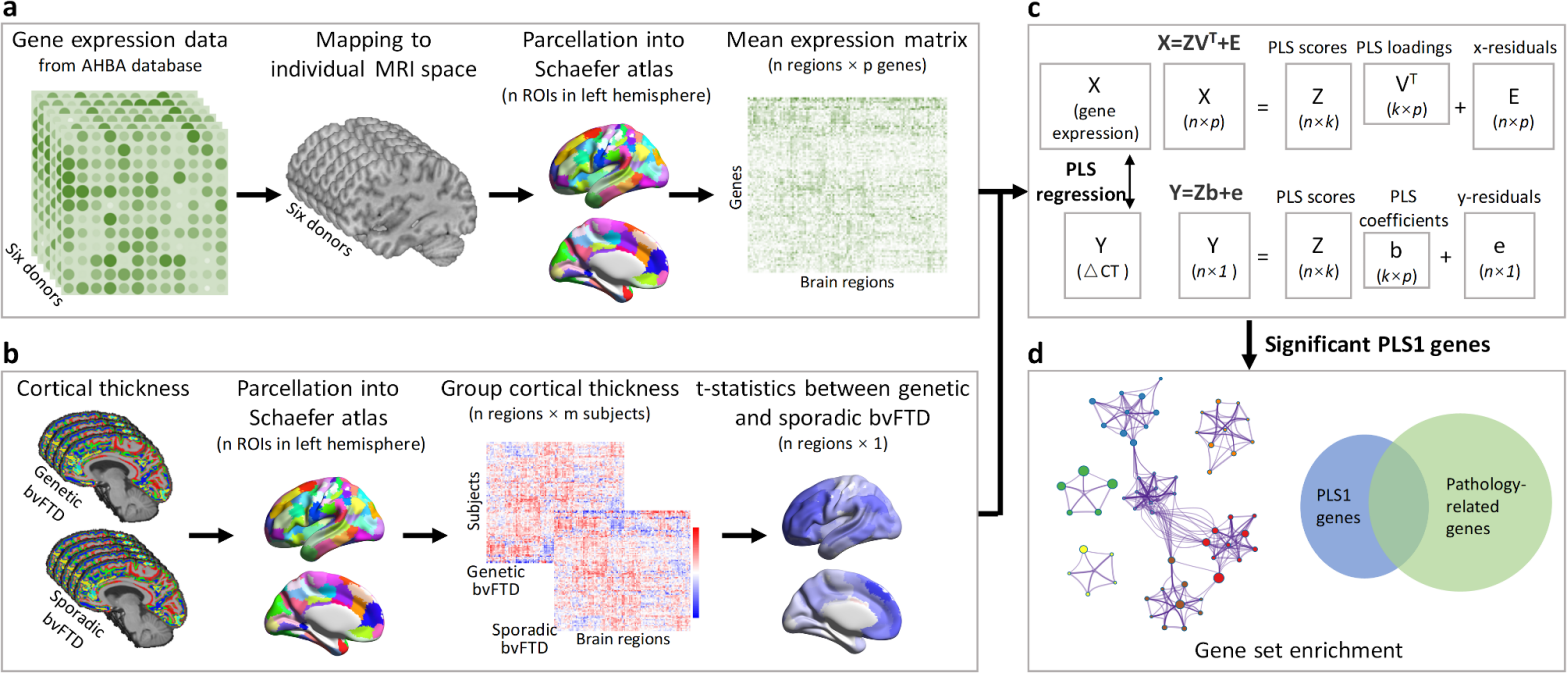
Overview of study pipeline. **a** Regional gene expression data extracted from the AHBA in 100 regions (left hemisphere only) across the 6 donors. **b** Cortical thickness data extracted from regions in the same atlas. The cortical thickness signatures were computed as t-statistic values compared between individuals with genetic and apparently sporadic bvFTD. **c** PLS regression was used to identify imaging transcriptomic associations. Genes with significant association were retained for subsequent analyses. **d** Functional enrichment analyses were conducted on genes with significantly loading on the PLS1, including functional enrichment and pathology enrichment analyses. AHBA = Allen Human Brain Atlas; PLS = partial least squares; PLS1 = first component of PLS

Here, we sought to bridge these gaps by relating transcriptomic data from the Allen Human Brain Atlas (AHBA) to macroscale structural neuroimaging in various forms of genetic bvFTD (*C9orf72*, *GRN*, and *MAPT*), relative to individuals with apparently sporadic bvFTD. Our goal was to explore potential molecular and cellular pathways that might explain regional vulnerability to neurodegeneration due to genetic pathogenic variants. The study framework is summarized in Fig. 1.

## Methods

### Participants

Participants were retrospectively selected from the Integrated Neurodegenerative Disease (INDD) database at University of Pennsylvania [11], consisting of 173 individuals with bvFTD, and 172 age, sex matched healthy controls who self-reported a negative neurological and non-significant psychiatric history with a normal Mini-Mental Status Examination (MMSE) > 27. Individuals with bvFTD were clinically diagnosed based on published criteria [1] and using a consensus procedure with > 2 clinical experts. Individuals with a concurrent diagnosis of amyotrophic lateral sclerosis (ALS), corticobasal degeneration, and progressive supranuclear palsy (PSP) were excluded.

Clinical and neuropsychological assessments were conducted at Penn Frontotemporal Degeneration Center. Neuropsychological test scores were obtained from the visit closest to MRI scan. Cognitive and behavioral changes were evaluated using MMSE and Philadelphia Brief Assessment of Cognition (PBAC) [12].

### Genetic screening

All participants underwent genetic evaluation including *C9orf72* repeat expansion testing, and sequencing and deletion/duplication analysis of *GRN* and *MAPT*. The genomic DNA was extracted from peripheral blood or frozen brain tissue samples [2]. The presence of a *C9orf72* repeat expansion was evaluated using a modified repeat-primed polymerase-chain reaction [13]. *GRN* and *MAPT* variants were identified from whole genome sequencing, whole exome sequencing, or targeted neurodegeneration sequencing panel datasets [11]. The bvFTD cases that were negative for *C9orf72* repeat expansions, or pathogenic variants in genes associated with ALS/FTD, were defined as sporadic bvFTD.

### Neuroimaging data acquisition and preprocessing

Structural T1-weighted MRI scans were acquired on a Siemens 3.0 Tesla scanner outfitted as a TIM Trio (*n* = 289) and subsequently as a Prisma Fit (*n* = 56). MRI scans were collected with magnetization-prepared rapid gradient-echo (MPRAGE) sequences as follows: (1) 3.0 Tesla Siemens TIM Trio scanner, 8 channel head coil, axial plane with repetition time (TR) ranging from 1620 ms to 1900 ms, echo time (TE) ranging from 3.09 ms to 4.38 ms, slice thickness = 1.0–1.5 mm, in-plane resolution = 0.98 × 0.98 mm. (2) 3.0 Tesla Siemens TIM Trio scanner, 64 channel head coil, sagittal plane with TR = 2200 ms or 2300 ms, TE ranging from 2.95 ms to 4.63 ms, slice thickness = 1.0–1.2 mm, in plane resolution = 1.0 × 1.0 mm. (3) 3.0 Tesla Siemens Prisma scanner, 64 channel head coil, sagittal plane with TR = 2400 ms, TE = 1.96 ms, slice thickness = 0.8 mm, in-plane resolution = 0.8 × 0.8 mm [14].

Images were processed using the Advanced Normalization Tools software through standard preprocessing steps [15], including N4 bias field correction, diffeomorphic and symmetric registration using joint label fusion to an aging brain template based upon 52 cognitively normal older adults from the Alzheimer’s Disease Neuroimaging Initiative (ADNI) Phase 1 (ADNI-1) study [16]. Template-based priors were used to inform brain extraction and segmentation into six-tissue classes (cortical gray matter, deep gray matter, white matter, CSF, brainstem, and cerebellum). This tissue segmentation was then used to estimate cortical thickness. The custom template was in turn aligned to the MNI152 2009c Asymmetric T1-weighted template. The Schaefer 7-network atlas with 200 cortical parcels [17] was then spatially normalized to each individual’s native space through the template-to-native warps generated by ANTs. Then cortical thickness was extracted from each label. To identify gene-specific atrophy patterns, we employed a w-score procedure to normalize cortical thickness values by age, and sex for each individual with bvFTD, relative to the mean distribution of 172 healthy controls. We then calculated the difference in w-scored cortical thickness for each cortical region between genetic and apparently sporadic bvFTD using a linear model, while adjusting for disease duration. Since only the left hemisphere was used, a 100 × 1 vector of case-control t-statistics for regional cortical thickness was obtained.

### Transcriptomic data and preprocessing

The AHBA is a publicly available resource providing whole-brain microarray gene expression data derived from post-mortem tissue samples of six adult human donors (https://human.brain-map.org/; Supplementary Table 1). This dataset includes expression profiles for over 20,000 genes, measured across 3,702 distinct tissue samples, representing the most spatially comprehensive “all genes, all structures” gene expression resource available for the human brain. We extracted transcriptomic data from six donors using abagen toolbox [18]. Only left hemisphere data were used, as right hemisphere samples were only available for two of the six donors. Transcriptomic data was processed according to previously described protocol including the following steps: probe-to-gene annotation, intensity-based filtering by a threshold of 0.5, probe selection by differential stability, donor aggregation in probe selection, matching tissue samples to the Schaefer 7-network atlas with 200 parcellation atlas [17], and data normalization. Finally, we obtained expression levels for 15,633 genes at 100 cortical regions, resulting in a 100 × 15,633 regional transcription matrix.

### Transcription-neuroimaging association analyses

Partial least squares (PLS) is a multivariate statistical method used to explore relationships between two datasets by identifying patterns that explain the most variance in both. It works by extracting components that represent shared variability between the two datasets, which are then used to make predictions or understand their relationships. In this analysis, a PLS regression model implemented in R (version 4.1.0) was used to investigate associations between cross-condition cortical thickness signatures in genetic bvFTD and brain regional gene expression. The regional transcription matrix (*X*, dimensions: 100 regions × 15,633 genes) was taken as predictor variables (Fig. 1a), and the regional cortical thickness signatures vector (*Y*, dimensions: 100 regions × 1) was treated as response variables (Fig. 1b). The PLS model decomposes *X* and *Y* into latent components (*Z*), weights (*V*), loadings (*V*^*T*^), regression coefficients (*b*), and residual terms (*E* and *e*):

*X* = *ZV*^*T*^ + *E*.

*Y* = *Zb* + *e*.

Here, *Z* (*n*×*k*) represents the shared latent variables across gene expression and cortical thickness signature, with *k* being the number of components. *V*^*T*^ (*k*×*p*) denotes PLS loadings for the genes, while *b* (*k*×1) refers to the PLS coefficients linking gene expression to cortical thickness. The residuals (*E* and *e*) capture variance not explained by the model (Fig. 1c). The first PLS component (PLS1), consisting of a linear combination of weighted gene expression values, that is most strongly associated with the anatomical map of cortical thickness. This component captures the largest shared variance between gene expression and cortical thickness. The variability and statistical significance of PLS1 weight for each gene was estimated by bootstrapping the cortical thickness vector (*Y*) 5,000 times (resampling with replacement of the 100 cortical regions) [19]. The ratio of the weight of each gene to its bootstrap standard errors was used to calculate z scores and then transformed to *p-values* to evaluate the contribution of each gene to PLS1. Only genes with *p-values* < 0.05 were regarded as significant contributors to PLS1 [20]. Significant genes with large positive or negative PLS1 weights were termed as PLS1 + or PLS1- gene sets.

### Functional enrichment analyses

We performed functional enrichment analyses to identify biological pathways associated with the PLS1 + and PLS1- gene sets separately using Metascape [21], focusing on 15,633 genes from the AHBA dataset as the background list. Metascape identifies gene ontology (GO) terms enriched with more genes from the input gene list than expected by chance, with significance defined as Benjamini-Hochberg false discovery rate (BH-FDR) corrected *q-value* < 0.05 [21]. Networks of enriched terms were visualized Cytoscape software to highlight similarities of these terms. Moreover, Metascape provides a Membership function that matches input genes to relevant GO terms retrieved based on specified keywords [21]. To investigate whether the PLS1 + or PLS1- genes were involved in specific neurotransmitter systems, we utilized this function to flag genes that were member genes of GO terms related to acetylcholine, dopamine, gamma-aminobutyric acid (GABA), glutamate, and serotonin systems.

### Mapping to neurotransmitter maps

To investigate the involvement of synaptic signaling in shaping brain structure in different genetic form of bvFTD, we downloaded PET scans from an assembled collection of about 1,200 normal subjects. These scans mapped the cortical distribution of 15 different receptors/transporters across five neurotransmitter systems, including acetylcholine, dopamine, GABA, glutamate, and serotonin [22]. PET images were acquired using various radioligands, resulting in different measured values, collectively referred to as density for simplicity [22]. All images were also registered to the MNI152 2009c Asymmetric T1-weighted template and parcellated into 200 regions according to the Schaefer 7-network atlas [17], generating group-averaged neurotransmitter density maps.

We performed spatial correlation analyses between neurotransmitter receptor/transporter densities maps and cortical thickness t-statistic map of each genetic form. Additionally, dominance analysis was used to evaluate the contribution of each predictor (15 neurotransmitter receptors/transporters) to cortical thickness signature using a multiple linear regression model [23]. The importance of each predictor was quantified by total dominance that summarizes the additional contributions of a predictor to all possible subset models, and by relative importance, expressed as a percentage of variance in the cortical thickness signature explained by each predictor.

### Pathology-related gene enrichment analyses

We hypothesized that the significant PLS1 genes identified in individuals with *C9orf72-*bvFTD and *GRN-*bvFTD were associated with TDP-43 pathology, and genes associated with *MAPT*-bvFTD were more involved in tau pathology. To explore whether the significant PLS1 genes were enriched for genes related to TDP-43 pathology or tau pathology, we extracted the genes from previously published studies [24, 25]. The genes related to TDP-43 pathology was defined by Liu and colleagues [24]. They fractionated and sorted for diseased neuronal nuclei from postmortem ALS-FTD human brains and identified 5,576 significantly differentially expressed genes (DEGs) that were associated with TDP-43 pathology. In terms of tau pathology, Allen and colleagues conducted correlation analyses between gene expression and a neuropathological latent trait of tau pathology in a PSP cohort to identify significant transcriptional associations. This resulted in identification of a list of 2,549 genes considered as tau pathology-related genes [25].

For each genetic form, we drew random sets of genes the same size as the significant PLS1 gene set, and calculated the number of overlapping genes between the random gene set and the TDP-43 or tau pathology-related gene lists. These procedures were repeated 1,000 times to obtain a null distribution of overlapping gene numbers. The null hypothesis was that the observed number of overlapping genes between PLS1-/+ gene sets and pathology-related gene lists was not significantly higher than the overlap between random gene set and pathology-related gene lists. The *p-value* of enrichment for TDP-43 or tau pathology-related genes were estimated by comparing the observed overlapped gene number to the null gene counts distribution. Therefore, significant gene overlap indicated enrichment for genes involved in TDP-43 or tau pathology.

### Shared transcriptional signatures across genetic forms

To explore the shared transcriptional signatures across different genetic forms, we focused on determining the extent of gene overlap between each pair of genetic forms by calculating the number of overlapping PLS1 genes present in each combination. These overlapping genes were then analyzed by conducting protein-protein-interaction (PPI) network analysis to predict functional partner genes provided by STRING database, encompassing both physical and functional associations [26]. Moreover, we proceeded to investigate the expression patterns of these overlapping genes across human tissues and cell types by leveraging data from the Human Protein Atlas (HPA) and genotype-tissue expression (GTEx) databases [27]. To portray gene expression levels, we extracted normalized transcript per million (nTPM) values, providing a quantitative representation of gene’s activity.

## Results

### Demographics and clinical characteristics

Table 1 displays the demographic and clinical features of 173 individuals with bvFTD, comprised of 117 individuals with apparently sporadic disease, 32 with *C9orf72* repeat expansions (> 30 repeats), 11 with pathogenic variants in *GRN*, and 13 with pathogenic variants in *MAPT* (Supplementary Table 2). Individuals with *MAPT*-bvFTD tended to be younger than other genetic forms or apparently sporadic cases. The individuals with *GRN*-bvFTD had a shorter disease duration compared to those with apparently sporadic bvFTD.

**Table 1:** Demographic and clinical characteristics of the study population

	Apparently sporadic (*n* = 117)	C9orf72 (*n* = 32)	GRN (*n* = 11)	MAPT (*n* = 13)	Missing data
Age at MRI (years)	62.3 (8.6)	62.7 (7.1)	62.1 (4.4)	54.0 (7.6)^a^	0.0%
Sex (male%)	72 (61.5%)	20 (62.5%)	6 (54.6%)	6 (46.2%)	0.0%
Disease duration (years)^b^	3.9 (2.7)	3.8 (2.7)	2.2 (1.2)^a^	3.6 (2.6)	0.0%
MMSE	24.1 (5.7)	23.9 (5.6)	21.3 (7.4)	22.8 (5.2)	12.1%
PBAC total	57.9 (15.6)	64.5 (13.2)	55.0 (16.0)	65.3 (13.7)	31.2%

Data are presented as mean (standard deviation) for the continuous variables, and as number (frequency) for the categorical variables.

^a^ indicates significant differences compared with the individuals with apparently sporadic bvFTD.

^b^ Disease duration is the interval between symptom onset and the time of scan.

bvFTD, behavioral variant frontotemporal dementia; MMSE, Mini-Mental Status Examination; PBAC, Philadelphia Brief Assessment of Cognition

### Cortical thickness signatures of different genetic forms of bvFTD

To identify cortical thickness signatures specific to genetic bvFTD, we compared the cortical thickness in each genetic group with that of apparently sporadic bvFTD (Supplementary Fig. 1). Individuals with *C9orf72*-bvFTD showed relatively spared cortical thickness in anterior and inferolateral temporal. *GRN*-bvFTD exhibited more extensive global reductions in cortical thickness, most predominant in inferior frontal cortex and parietal regions including precuneus and posterior cingulate cortex. *MAPT*-bvFTD was associated with globally reduced cortical thickness with more predominantly reduced cortical thickness in insula, motor cortex, and dorsolateral prefrontal cortex.

### Gene expression patterns associated with cortical thickness signatures

**Fig. 2:**
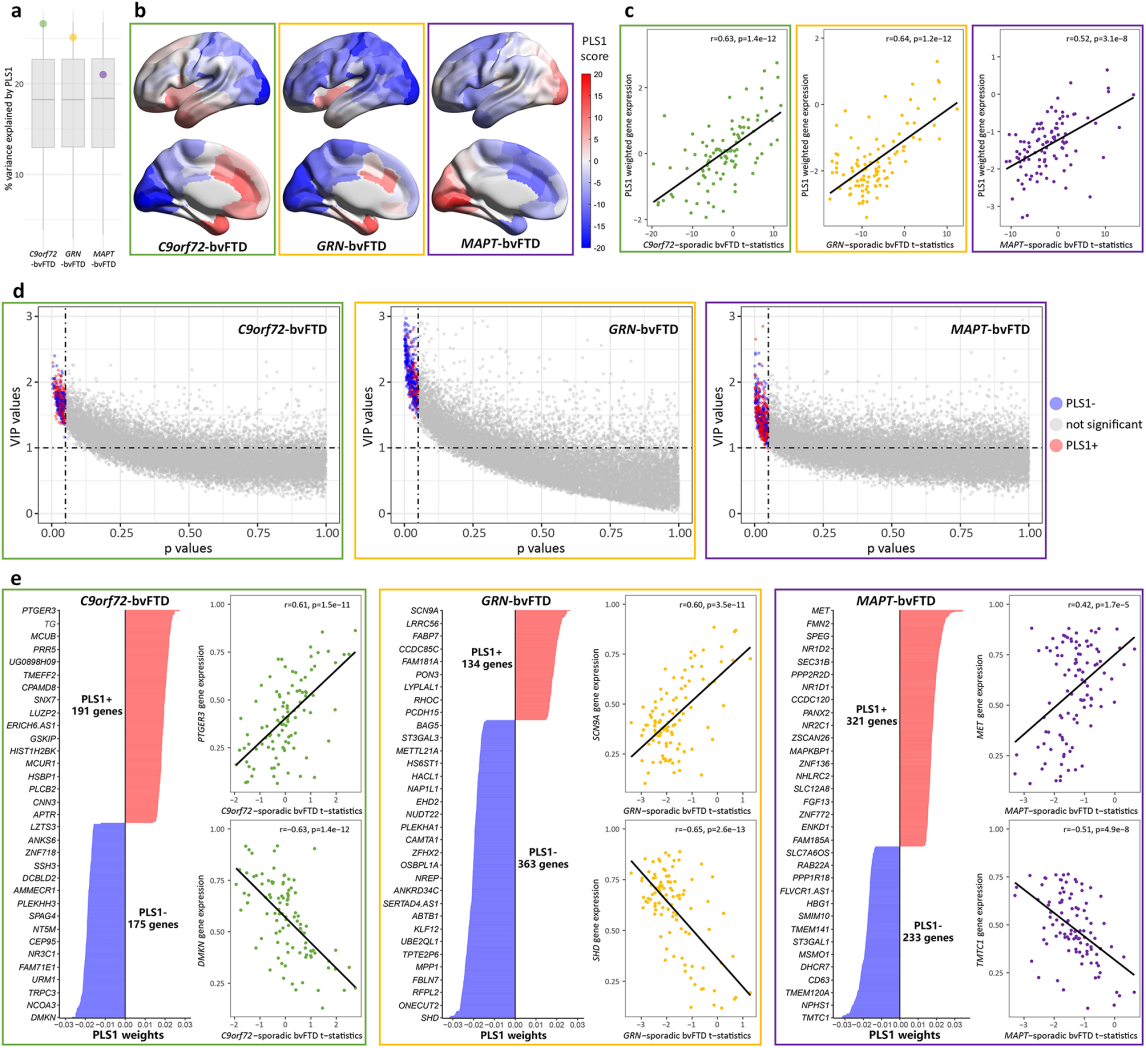
Gene expression patterns associated with cortical thickness signatures.**a** The percentage of the variance in cortical thickness signature explained by PLS1. The boxplots represent the null distribution of variance explained by PLS1 obtained through 5,000 bootstrap iterations, while each dot corresponds to the observed value. **b** PLS1 weighted gene expression of each genetic form of bvFTD. Darker red indicates higher PLS1 scores, whereas darker blue corresponds to lower scores. **c** Significantly positive correlations between PLS1 weighted gene expression and anatomical map of regional cortical thickness signatures. **d** Genes with a VIP score > 1, and bootstrapping *p value* < 0.05 were selected as top-ranked genes. Blue dots represent genes with expression levels negatively correlated with cortical thickness, red dots represent genes with expression levels positively correlated with cortical thickness, and gray dots represent genes with no significant correlation. **e** Bar plots display genes ranked by their PLS1 weights. The weights are coefficients of the linear combination of the original variables used to predict the response variables in the PLS regression model. Scatter plots show correlations between the anatomical map of regional cortical thickness signatures and gene expression level of the top positively or negatively weighted genes. PLS = partial least squares; PLS1 = the first component of PLS; VIP = importance in projection

We used a PLS regression model to examine the brain spatial correlation between gene expression and cortical thickness signatures. PLS1 + genes were overexpressed in regions where cortical thickness increased, while PLS1- genes have increased expression in regions where the cortical thickness decreased. This procedure was performed separately for each genetic form of bvFTD. All significant genes were shown in Supplementary Table 3. For *C9orf72*-bvFTD, PLS1 explains 26.6% of the variance in cortical thickness signature, which is greater than chance (*p*_*boot*_ = 0.001; Fig. 2a). The weighted gene expression map of PLS1 exhibited a gradient of gene expression levels, ranging from higher gene expression in left-predominant anterior temporal, insular, and anterior cingulate regions and lower gene expression associated with the more spared parietal-occipital cortex (Fig. 2b). The gene expression level, reflected by the PLS1 score correlated with the regional t-statistic map of cortical thickness (Fig. 2c). Genes with a variable importance in projection (VIP) > 1 [28] and *p-values* < 0.05 were considered as significant contributors (Fig. 2d). We identified 191 PLS1 + and 175 PLS1- genes for *C9orf72*-bvFTD (Fig. 2e). In *GRN*-bvFTD, we observed that the PLS1 explained 25.1% of the variance (*p*_*boot*_ = 0.08; Fig. 2a). PLS1 weighted gene expression map also displayed a pattern of higher gene expression in anterior regions and lower gene expression in posterior regions (Fig. 2b). We identified 134 PLS1 + and 363 PLS1- genes for this group (Fig. 2e). Regarding the *MAPT*-bvFTD, cortical atrophy was accounted by PLS1 to an extent of approximately 21.0% (*p*_*boot*_ = 0.36; Fig. 2a). Notably diverging from the patterns observed in *C9orf72*-bvFTD and *GRN*-bvFTD, a distinct feature of this group was the presence of a posterior-anterior gradient in the distribution of gene expression (Fig. 2b). We identified 321 PLS1 + and 233 PLS1- genes for *MAPT*-bvFTD (Fig. 2e).

### Functional enrichment analyses

**Fig. 3:**
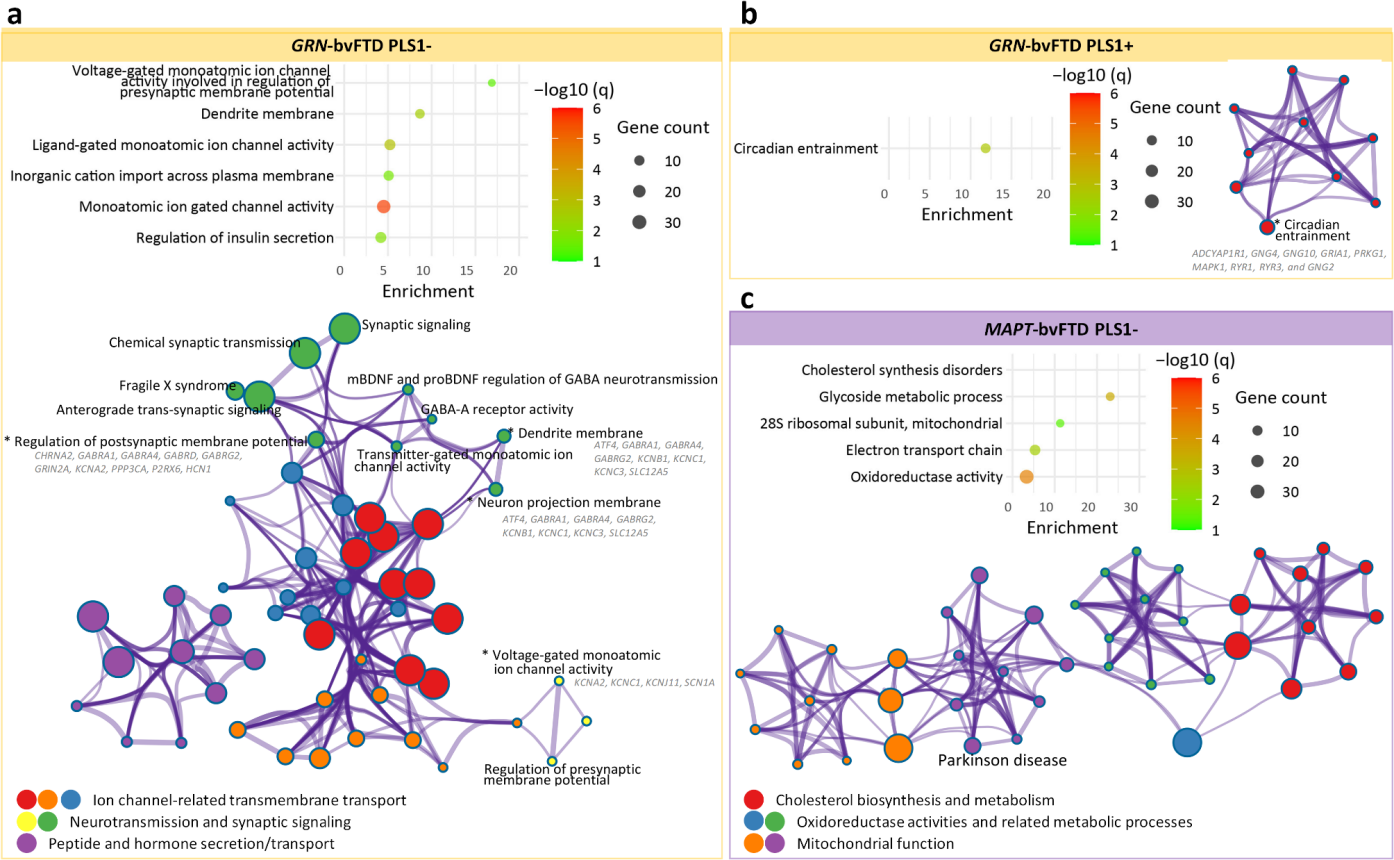
Results of functional enrichment analyses. **a-b** Results of functional enrichment of PLS1- (**a**) and PLS1+ (**b**) genes associated with cortical thickness signatures of *GRN*-bvFTD. **c** Results of functional enrichment of PLS1- genes associated with cortical thickness signatures in *MAPT*-bvFTD. All significant resultant terms were clustered into groups based on their similarities. The bubble plot shows the subset of representative ontology terms for each enriched cluster. The bubble size represents the overlapping gene count between the PLS1 gene set and genes involved in each ontology term. The color bar represents the logarithmically transformed BH-FDR-corrected *q values*. Metascape enrichment network visualization showing the intra-cluster and inter-cluster similarities of enriched terms. Nodes are colored to reflect their cluster memberships. * indicates that the biological processes were significant. Genes that involved in these significant terms were listed in the figure. BH-FDR = Benjamini-Hochberg false discovery rate

We performed functional enrichment analyses on PLS1 + and PLS1- gene lists separately (Supplementary Table 4). The PLS1-/+ gene sets associated with *C9orf72*-bvFTD did not exhibit statistically significant enrichments. However, the PLS1 + gene list showed connections with biological processes including “neurotransmission and synaptic signaling”, “neural development and structure”, and “perception”. The PLS1- genes of *GRN*-bvFTD were significantly enriched for neural-related terms, such as “neuronal membrane components” and “regulation of synaptic membrane potential”, as well as biological processes related to “ion channel-related transport” (Fig. 3a). The PLS1 + genes of *GRN*-bvFTD were significantly enriched in “circadian entrainment” (Fig. 3b), and also have connections with “neurotransmission and synaptic signaling”, although the latter did not reach statistical significance. Both PLS1- and PLS1 + gene lists of *GRN*-bvFTD were associated with GABAergic synapse, with PLS1- genes additionally linked to dopaminergic synapse, while PLS1 + genes linked to glutamatergic, cholinergic, and serotonergic synapses. The PLS1- genes of *MAPT*-bvFTD were enriched in biological terms related to “cholesterol biosynthesis and metabolism”, “mitochondrial function”, and “oxidoreductase activities”, which were involved in various systems, not specific to nervous system (Fig. 3c). Additionally, this gene set tended to be associated with pathways related to Parkinson’s disease (PD). While not reaching statistical significance, the PLS1 + genes of *MAPT*-bvFTD was also associated with “circadian rhythm” and “regulation of behavior”.

### Pathology-related gene enrichment analyses

**Fig. 4:**
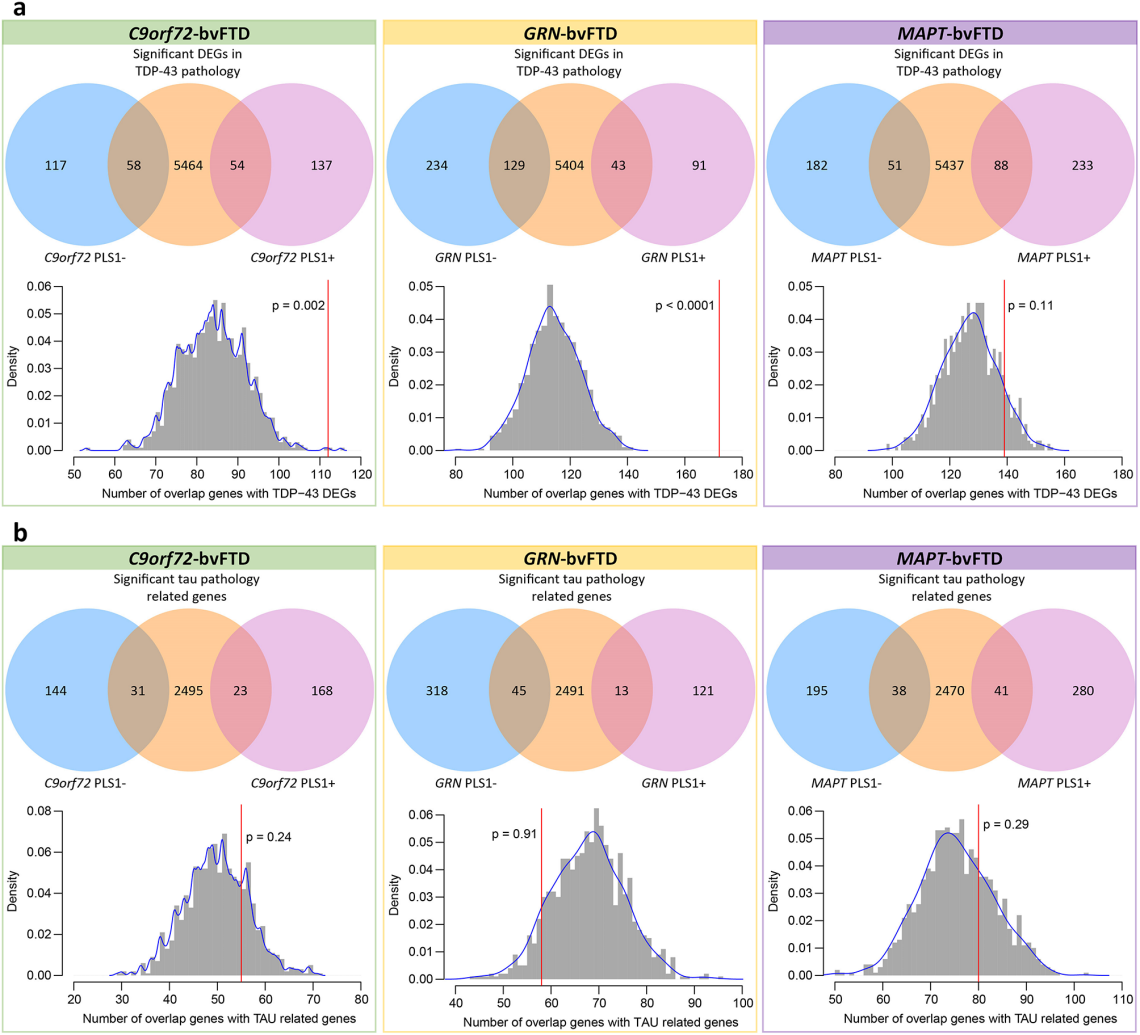
Pathology-related enrichment analyses. **a-b** The overlap between PLS1 gene lists associated with each genetic form of bvFTD and TDP-43 pathology-related DEGs (**a**) and tau pathology-related genes (**b**). Venn diagrams (upper) showing the overlap between groups. Histograms (bottom) display the null distribution, depicting the number of overlapped genes between PLS1 genes and pathologies related genes. The red line represents the observed number of overlapped genes. PLS1 = first component of partial least squares; DEGs = differentially expressed genes

For TDP-43 pathology, we used a gene list comprised of DEGs observed in fluorescent-activated cell sorting (FACS) sorted TDP-43 positive compared to TDP-43 negative neurons [24], our findings revealed that PLS1 genes of *C9orf72*-bvFTD and *GRN*-bvFTD groups were enriched in these genes linked to TDP-43 pathology, whereas PLS1 genes of *MAPT*-bvFTD did not show significant enrichment (Fig. 4a). There were 112 genes associated with *C9orf72*-bvFTD, 172 genes with *GRN*-bvFTD, and 139 genes with *MAPT*-bvFTD intersecting with the TDP-43-related genes (Supplementary Table 5). Notably, these shared genes in *C9orf72*-bvFTD and *GRN*-bvFTD groups significantly encompass crucial components involved in neural pathways of “neurotransmission and synaptic signaling” (Supplementary Table 6).

In the analysis of tau pathology in PSP, a previous study identified a gene set significantly associated with the overall burden of tau pathology [25]. However, we did not observe significant enrichment of the three genetic forms in this tau pathology-related gene set (Fig. 4b). There were 55 genes associated with *C9orf72*-bvFTD, 58 genes with *GRN*-bvFTD, and 80 genes with *MAPT*-bvFTD intersecting with the tau pathology-related gene set. (Supplementary Table 5). While not reaching statistical significance, these shared genes were mainly associated with biological processes that were involved in various systems, such as “transcriptional regulation and protein processes”, “cellular processes and organization”, and “ion transport and signaling” (Supplementary Table 6), rather than pathways highly specific to nervous system.

### Mapping to synaptic density maps

**Fig. 5:**
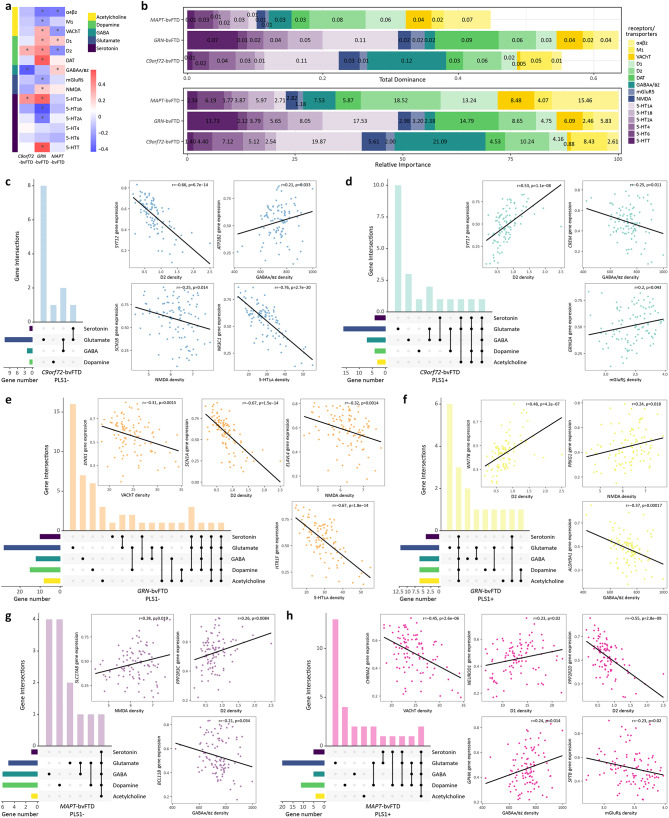
Mapping synaptic density maps to cortical atrophy and gene expression patterns. **a** Correlations between cortical thickness t-statistic maps and neurotransmitter receptor/transporter density maps. * indicates that the correlation is significant. **b** Results of dominance analysis. Upper bar plot displays the importance of 15 neurotransmitter receptor/transporter densities in predicting cortical atrophy. Lower bar plot illustrates the relative importance of each variable. **c-h** Upset plots illustrate genes involved in five neurotransmitter systems. Horizontal bars show the total number of genes involved in each neurotransmitter system. Bottom matrix indicates neurotransmitter systems involved in each intersection. Filled dot indicates the presence of a system in that intersection, and connected dots indicate a combination of systems in that intersection. Scatter plot shows the correlation between the expression map of a certain gene and a neurotransmitter density map

After identifying gene sets associated with “neurotransmission and synaptic signaling”, we correlated the density maps of five neurotransmitter systems with cortical thickness t-statistic map of each bvFTD genetic form (Fig. 5a). *C9orf72*-bvFTD t-statistic map positively correlated with dopaminergic (D_2_ receptor) and serotonergic (5-HT1_A_ receptor) neurotransmitter densities, while negatively correlated with GABAergic neurotransmitter receptor density. *GRN*-bvFTD t-statistic map showed positive correlations with dopaminergic neurotransmitters, while showed either positive or negative correlations with different receptors of other neurotransmitter systems. *MAPT*-bvFTD t-statistic map positively correlated with GABAergic neurotransmitter receptor density, negatively related to cholinergic neurotransmitter density, and showed opposing correlations with dopaminergic neurotransmitters, being positive for D_1_ and negative for D_2_ receptor density.

Dominance analysis, which consider the correlations between variables, were conducted to evaluate the importance of the 15 neurotransmitter receptors/transporters in relation to cortical atrophy in different genetic forms of bvFTD (Fig. 5b). The serotonergic (5-HT1_A_ receptor) and GABAergic neurotransmitter systems were the most important variables relating to cortical thickness in *C9orf72*-bvFTD. In *GRN*-bvFTD, the serotonergic (5-HT1_A_ and 5-HTT receptors) and dopaminergic (dopamine transporter) neurotransmitter systems were the most important variables. In *MAPT*-bvFTD, dopaminergic (D_1_ and D_2_ receptors) and cholinergic (α_4_β_2_ receptor) neurotransmitter systems showed the highest importance in relation to cortical thickness.

Membership analyses revealed that each PLS1 gene set contains genes belonging to GO terms of these neurotransmitter systems, with gene expression maps correlating to neurotransmitter receptor/transporter density maps (Fig. 5c-h and Supplementary Fig. 2). Higher proportion of genes in *MAPT*-bvFTD PLS1- list fell into dopaminergic and GABAergic terms, while other gene lists were more related to glutamatergic system. For *GRN*-bvFTD, the terms identified in membership analysis involved five neurotransmitters, and were significantly enriched in PLS1-/+ gene lists (Supplementary Fig. 3). These findings are consistent with correlations observed between *GRN*-bvFTD cortical thickness t-statistic map and neurotransmitter maps, further support the involvement of neurotransmission and synaptic signaling in *GRN*-related brain atrophy.

### Shared genes across genetic forms of bvFTD

**Fig. 6:**
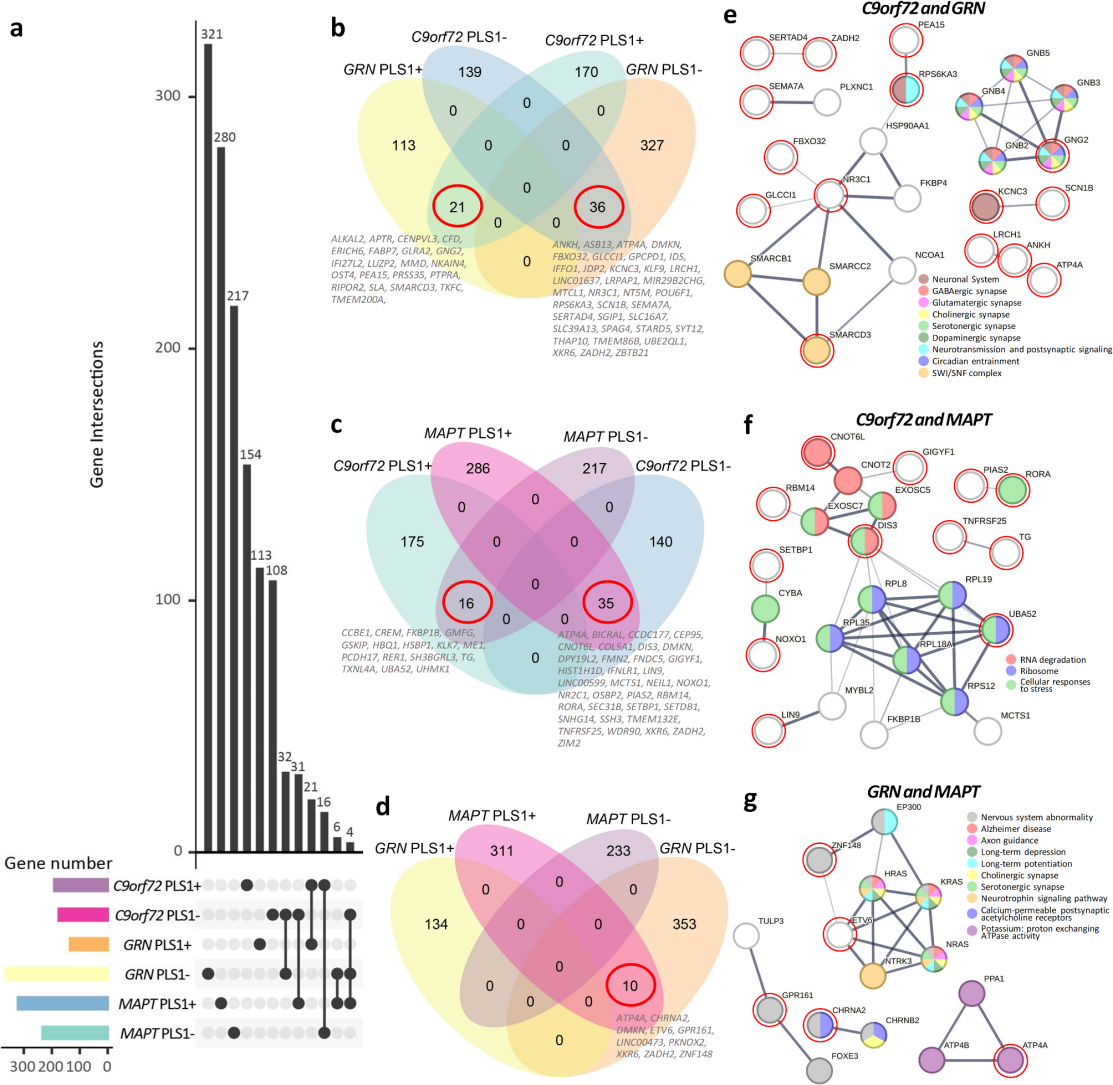
Shared genes across genetic forms of bvFTD. **a** Upset plot illustrate the gene intersections across groups. **b** Venn diagrams showing the genes that associated with cortical thickness were shared between *C9orf72*-bvFTD and *GRN*-bvFTD. **c** Shared genes between *C9orf72*-bvFTD and *MAPT*-bvFTD. **d** Shared genes between *GRN*-bvFTD and *MAPT*-bvFTD. **e-j** STRING interaction diagram of the overlapped genes. Each node denotes a gene, edges between nodes indicate interactions between protein products of the corresponding genes, with the line thickness indicating the strength of interactions. Genes are colored according to the functional pathways that these genes involved. The genes circled in red represent the overlapped genes that we identified, while the genes without circles are predicted functional partners provided by STRING

Despite different genetic sources, bvFTD presents as a relatively homogenous syndrome, suggesting that there may be shared genetic factors influencing brain structures across various genetic forms. We further identified shared transcriptomic signatures across three genetic forms (Fig. 6a). *C9orf72*-bvFTD and *GRN*-bvFTD had several overlapping genes with consistent directionality (Fig. 6b). Shared genes with positive weights in *C9orf72*-bvFTD also displayed positive weights in *GRN*-bvFTD, indicating their common expression in regions more spared from cortical atrophy. Conversely, shared genes with negative weights in *C9orf72*-bvFTD also displayed negative weights in *GRN*-bvFTD, suggesting that these genes were commonly expressed in regions more vulnerable to brain atrophy. *MAPT*-bvFTD had overlapping genes with the other two forms (Fig. 6c-d), all with opposing directionality. This indicates that these shared genes had opposite correlations with cortical thickness in *C9orf72*-bvFTD and *GRN*-bvFTD compared to *MAPT*-bvFTD.

We constructed PPI networks on these overlapping genes using STRING database (Fig. 6e-j). Among the overlapping genes between *C9orf72*-bvFTD and *GRN*-bvFTD, G protein subunit gamma 2 (*GNG2*) was involved in synaptic signaling and circadian entrainment. The potassium voltage-gated channel subfamily C member 3 (*KCNC3*) and ribosomal protein S6 kinase alpha-3 (*RPS6KA3*) genes may contribute to other aspects of neurotransmission. The SWI/SNF-related, matrix-associated, actin dependent regulator of chromatin, subfamily d, member 3 (*SMARCD3*) gene encodes a protein belonging to SWI/SNF family, involving in regulating gene transcription through chromatin remodeling. The shared genes with opposing directionality in *GRN*-bvFTD and *MAPT*-bvFTD included the cholinergic receptor nicotinic alpha 2 subunit (*CHRNA2*), G protein-coupled receptor 161 (*GPR161*), and zinc finger protein 148 (*ZNF148*) that were linked to nervous system abnormalities. The ETS Variant Transcription Factor 6 (*ETV6*) gene was connected to pathways involving synaptic signaling and axon guidance. Overlapping genes with opposing directionality in *C9orf72*-bvFTD and *MAPT*-bvFTD directly or indirectly associated with RNA degradation, ribosome function, and cellular responses to stress.

**Fig. 7:**
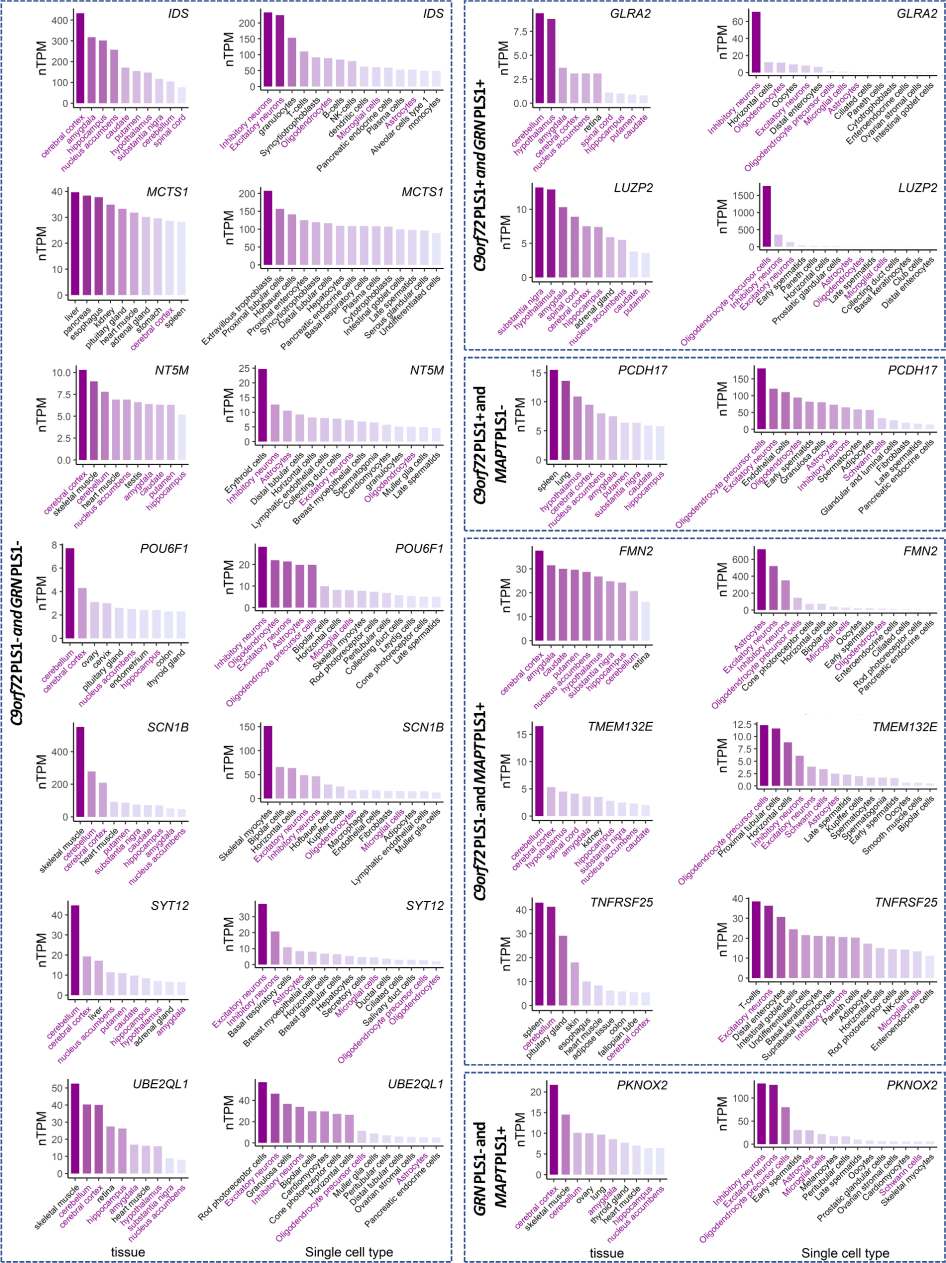
The expression level of overlapping genes in multiple tissues and cell types. Only genes specifically enriched in both brain tissues and neural cells were shown

Among the overlapping genes, the glycine receptor alpha 2 (*GLRA2*), leucine zipper protein 2 (*LUZP2*), iduronate 2-sulfatase (*IDS*), microtubule crosslinking factor 1 (*MTCL1*), 5’,3’-nucleotidase, mitochondrial (*NT5M*), POU class 6 homeobox 1 (*POU6F1*), sodium voltage-gated channel beta subunit 1 (*SCN1B*), synaptotagmin 12 (*SYT12*), ubiquitin conjugating enzyme E2 Q family like 1 (*UBE2QL1*), protocadherin 17 (*PCDH17*), formin 2 (*FMN2*), transmembrane protein 132E (*TMEM132E*), TNF receptor superfamily member 25 (*TNFRSF25*), and PBX/knotted 1 homeobox 2 (*PKNOX2*) genes, were specifically enriched in brain and neural cells, as indicated by GTEx database and single cell type expression maps in HPA database (Fig. 7). Additionally, there were 17 other genes that showed specific enrichment in brain neural cells, while 14 genes displayed specific enrichment in brain tissues (Supplementary Fig. 4).

## Discussion

This study employed an integrative imaging-transcriptomic approach to uncover the transcriptomic signatures associated with cortical thinning across various genetic subgroups of bvFTD. We observed distinct patterns of cortical thinning in each genetic form of bvFTD compared to apparently sporadic bvFTD. Subsequently, we utilized a PLS regression model to identify genes whose expression levels across brain regions exhibited strong correlations with cortical thinning. Our findings suggested the presence of distinct molecular mechanisms specific to different genetic forms, and shared molecular mechanisms contributing to cortical remodeling in different genetic forms.

To establish convergence with prior findings on genetic bvFTD [4, 6, 29, 30], we first identified cortical signatures for each group. Diverse but overlapping brain atrophy patterns have been identified among various genetic forms of bvFTD [4], and our findings provide additional supporting evidence. In our study, individuals with *C9orf72*-bvFTD exhibited higher cortical thickness in temporal lobe. This observation aligns with previous findings indicating that individuals with *C9orf72* repeat expansions rarely present with semantic variant primary progressive aphasia (svPPA), which is a temporal-predominant syndrome [3, 29]. Moreover, FTLD-TDP type A and type B are predominant subtypes that associated with *C9orf72*, were more spared in temporal cortex [31, 32]. For *MAPT*-bvFTD, we observed greater grey matter loss in temporal pole and prefrontal cortex, consistent with previous reports of temporal and orbitofrontal lobe atrophy in this genetic form [30]. In contrast, *GRN*-bvFTD exhibited more extensive cortical atrophy, affecting various brain regions including prefrontal, temporal, somatomotor, visual, posterior cortices, precuneus and posterior cingulate. Individuals with *GRN*-bvFTD also demonstrated later disease onset, shorter disease duration, and faster atrophy progression. This suggested that they may have a prolonged latent phase, followed by rapid disease progression once a critical threshold is reached. Previous study also reported that *GRN*-FTLD experienced faster atrophy progression compared to other genetic forms [6]. These observed patterns may not necessarily reflect the overall clinical phenotype of bvFTD, but could be solely attributed to the pathogenic variants.

Using a PLS regression model, we identified significant transcriptional associations with neuroimaging. Our analysis revealed distinct sets of PLS1 genes positively or negatively associated with cortical atrophy for *C9orf72*-bvFTD, *GRN*-bvFTD, and *MAPT*-bvFTD, potentially reflecting molecular mechanisms underlying neuroanatomical changes. Distinct spatial gene expression patterns specific to each genetic form provide valuable insights into the molecular mechanisms that may influence cortical remodeling and underlie the disease heterogeneity across different genetic forms of bvFTD. For the identified PLS1 genes, enrichment analysis highlighted their biological significance and pathological relevance, focusing on synaptic systems, circadian dysregulation, and TDP-43/tau pathology. Differences and similarities among the three genetic forms were discussed in each of these contexts.

Extensive frontotemporal synaptic loss has been documented in bvFTD, correlating with disease severity [33]. BvFTD is a major clinical subtype of FTLD [1], associated with alterations in various neurotransmitter systems, including glutamatergic, GABAergic, dopaminergic, and serotonergic systems [34], constituting a fundamental molecular mechanism underlying FTD. Previous studies also had shown widespread involvement of these neurotransmitter systems across various genetic subgroups of FTD [35]. Among genes that positively associated with cortical thickness signature of *C9orf72*-bvFTD, there were certain genes holding notable functional importance in “neurotransmission and synaptic signaling”, with a specific emphasis on GABAergic systems. By mapping different neurotransmitter systems to cortical structure, we further confirmed that GABAergic signaling pathways contributed the most in predicting brain atrophy in individuals with *C9orf72*-bvFTD, which was also supported by findings that genes associated with GABAergic signaling are impacted in *C9orf72*-FTLD [36]. Additionally, there also existed genes related to “neural development and structure”, such as the cytoplasm of neuron projections (axons). As widely recognized, repeat expansions in *C9orf72* triggers abnormal RNA-processing events, crucially impacting the organization of synapses and their cytoskeletal components, as well as modulating synaptic strength and function [37]. Similarly, within the genes associated with cortical atrophy in *GRN*-bvFTD, there were genes connecting to “neurotransmission and synaptic signaling”. Both the negatively and positively associated gene sets encompassed genes related to GABAergic system. Furthermore, negatively associated genes were related to dopaminergic synapse, whereas the positively associated genes were linked to glutamatergic, cholinergic, and serotonergic synapses. By mapping different neurotransmitter systems to cortical structure, the involvement in *GRN*-bvFTD spans multiple neurotransmitter systems, thereby reinforcing the role of various neurotransmitter pathways in cortical atrophy in *GRN*-bvFTD. Our findings align with previous studies, as pathogenic variants in *GRN* may impact synaptic function, such as impairments in synaptic connectivity, plasticity, and transmission [38]. In *MAPT*-bvFTD, cholinergic, dopaminergic and GABAergic neurotransmitters are important contributors in predicting brain atrophy. Individuals with pathogenic variants in *MAPT* gene were observed with dopaminergic dysfunction [39]. Just as the haplotype of *MAPT* gene is linked to PD [40], several genes we have identified could also potentially be involved in biological processes related to PD that strongly associated with dopaminergic neurotransmission. Moreover, *MAPT* pathogenic variants may trigger alterations in GABAergic signaling and synaptic function, leading to pathogenesis of tauopathies [41]. In general, these observations further highlight the critical role of neurotransmission and synaptic signaling in cortical remodeling across different genetic forms, with distinctions arising in the specific aspects of synaptic systems they affect. Notably, these relationships are more extensive in *GRN*-bvFTD.

Circadian dysregulation is increasingly recognized as an early and progressive feature of bvFTD [42]. Neurodegenerative diseases are linked to pathological disruptions in circadian and sleep regulatory networks that encompass brain regions such as hypothalamus, basal forebrain, and mesial temporal lobe [42]. In our study, both *MAPT*-bvFTD and *GRN*-bvFTD groups exhibited genes involved in “circadian” pathways. Moreover, there are genes associated with *C9orf72*-bvFTD were specifically expressed in cholinergic neurons in basal forebrain and hypocretinergic neurons in hypothalamus. Notably, these regions and pathways play pivotal roles in regulation of circadian system [43]. However, circadian dysfunction may be more pronounced in *MAPT*-FTD. Sleep disturbances in these three genetic forms of FTD could be linked to atrophy in sleep-relevant hypothalamic subregions, with *MAPT*-FTD showing the most severe and consistent deficits [44]. Proteinopathies such as tauopathy have the potential to disrupt circadian rhythm. Conversely, circadian dysregulation also influences the occurrence and progression of these pathologies [45, 46]. One pathology study reported a greater tau burden compared to TDP-43 burden in locus coeruleus, a region implicated in circadian functions [47], which also suggested that *MAPT* carriers may have a more pronounced impact. Circadian rhythmicity also involves the regulation of glymphatic system, a brain waste clearance system responsible for eliminating proteins and other solutes from interstitial fluid [48]. Circadian dysregulation may potentially lead to glymphatic system failure, impeding clearance of abnormal protein accumulations such as TDP-43 and tau proteins. Glymphatic dysfunction could serve as a common pathway towards various forms of dementia [48]. Thus, this shared involvement in circadian-related pathways may represent common pathological regulators of these genetic forms. However, specific genes within these pathways vary due to distinct genetic backgrounds.

We also investigated the correlation between these transcriptional features and different proteinopathies. As we expected, consistent with the widely acknowledged phenomenon that *C9orf72*-bvFTD or *GRN*-bvFTD typically exhibit TDP-43 pathology [49], the gene sets associated with cortical thickness signature of both *C9orf72*-bvFTD and *GRN*-bvFTD showed significant enrichment for TDP-43 pathology-related DEGs. Conversely, *MAPT* variants predominantly lead to tau pathology, resulting in absence of TDP-43 pathology enrichment within the gene sets related to *MAPT*-bvFTD. The identification of these overlapping genes between *C9orf72*-bvFTD or *GRN*-bvFTD and TDP-43 pathology, provides valuable insights into the shared molecular pathways that might underlie their potential involvement in disease pathogenesis. We further examined the enrichment of tau pathology in the context of PSP. The gene sets associated with *C9orf72*-bvFTD and *GRN*-bvFTD did not yield significant results in this regard. This underscores the pathology-specific nature of these genes, aligning with the pathological presentations of these two genetic forms. However, genes associated with *MAPT*-bvFTD did not display significant enrichment for PSP-tau pathology. This could potentially be attributed to the fact that these tau-related genes were identified in PSP that link to 4R tauopathy, while FTLD-tau owing to different *MAPT* variants can result in 3R, 4R or mixed 3R/4R tauopathies [3]. Moreover, genes associated with *MAPT*-bvFTD were enriched in pathways not directly linked to nervous system, including “cholesterol biosynthesis and metabolism”, “mitochondrial function”, and “oxidoreductase activities”. This could also explain the lack of significant enrichment for tauopathy in this genetic form.

We further delved deeper into the shared genes identified across *C9orf72*-bvFTD, *GRN*-bvFTD, and *MAPT*-bvFTD, indicating common molecular foundations among different genetic forms. Both *C9orf72*-bvFTD and *GRN*-bvFTD that linked to TDP-43 pathology [50], their transcriptomic signatures were enriched in previously identified genes linked to TDP-43 positive neurons. These two genetic forms shared genes displaying consistent directionality, with those exhibiting either positive or negative correlations with cortical thickness in *C9orf72*-bvFTD showing the same direction (positive or negative) in *GRN*-bvFTD. *MAPT*-bvFTD that associated with tauopathy [50], displayed more pronounced transcriptomic differences, since *MAPT*-bvFTD had shared genes with the other two forms, but with opposing directionality. These shared genes that are commonly expressed in regions more spared by cortical atrophy in *C9orf72*-bvFTD and *GRN*-bvFTD, and conversely, these genes are commonly expressed in regions more vulnerable to brain atrophy in *MAPT*-bvFTD. Thus, the shared transcriptomic signatures exhibited similar relationships with cortical remodeling due to pathogenic variants in *C9orf72* and *GRN*, while showed opposite relationships with cortical remodeling attributed to pathogenic variants in *MAPT*.

Moreover, our focus further centered on several key genes within this shared gene pool. One prominent gene is *GNG2*, which was shared by *C9orf72*-bvFTD and *GRN*-bvFTD, in collaboration with other genes such as guanine-nucleotide binding proteins (*GNB2*, *GNB3*, *GNB4*, and *GNB5*), is known to be intricately involved in driving synapse-related pathways [51]. Another key gene is *SMARCD3*, in conjunction with *SMARCB1* and *SMARCC2*, encodes proteins belonging to the SWI/SNF family, which play crucial roles in regulating gene transcription through chromatin remodeling, thereby impacting neural development, proliferation and degeneration [52, 53]. The *EVT6* gene presented in *GRN*-bvFTD and *MAPT*-bvFTD, might not exert a direct influence, but its interaction with RAS genes like *NRAS*, *KRAS*, and *HRAS* has implications for synaptic signaling and axon guidance. Additionally, the *CHRNA2* gene, along with *CHRNB2* gene, is associated with synaptic signaling processes. These findings illuminate key genetic factors contributing to the shared molecular mechanisms across genetic forms of bvFTD, enhancing our understanding of complex interplay within neurodegeneration.

There are several limitations to consider in future work. One limitation is the utilization of regional transcriptomic data from only six donors. We excluded data from the right hemisphere due to limited availability, potentially introducing biases related to asymmetric cortical atrophy and gene expression patterns across two hemispheres. This constraint reflects a broader challenge in the field, as acquiring a comprehensive whole-brain atlas of gene expression for all genes across all regions remains challenging. Secondly, we correlated this constitutive gene expression data with neuroimaging features derived from a different cohort. While it was assumed that regional gene expression serves as a conserved canonical signature and its generalizability extends far beyond these 6 brains [54, 55], this remains a noteworthy limitation that lies in the potential incomplete representation of our cohort by this regional gene expression atlas. In addition, the PLS regression model requires the same number of samples for predictor (e.g. transcriptome) and response (e.g. neuroimaging) variables, and in our case, the samples correspond to brain regions. Gene expression for each region was averaged across donors, while cortical thickness signatures were the statistical differences between genetic and apparently sporadic bvFTD. Thus, sample size can influence the averaged gene expression and estimates of cortical thickness signatures, thereby influencing the results [56]. Moreover, the relatively small sample size in this study likely amplifies inter-individual variability within each genetic group. This variability may arise from differences in disease progression stages, methodological inconsistencies, or unmeasured environmental factors, potentially affecting the cortical thickness signatures and the correlations observed in the PLS regression model. Therefore, larger cohorts are essential for validating these findings and minimizing the influence of inter-individual variability. Additionally, the spatial colocalization between gene expression and cortical atrophy does not necessarily imply a causal relationship between them. The identified genes require validation through forthcoming animal experiments to assess their functions and impact. Furthermore, in the investigation of enrichment in tauopathy-related genes, we utilized a PSP dataset due to the limited studies with comprehensive transcriptomic data specifically examining tau pathology in bvFTD. While both PSP and bvFTD share tau pathology, the distinct spatial topology of tau deposition in these diseases may introduce variability. As such, further studies specifically addressing tau pathology in bvFTD are needed to refine our understanding of these distinct conditions.

## Conclusions

Overall, we identified both disparate and shared transcriptomic signatures associated with cortical atrophy in bvFTD with pathogenic variants in *C9orf72*, *GRN*, and *MAPT*. The spatial distribution of gene expression may contribute to selective vulnerability of macroscopic neuroanatomy, giving rise to the heterogeneity observed in bvFTD. Our study provided evidence mapping genetic-related variance in cortical thickness to synaptic genes, circadian-related genes and dysregulated genes in TDP-43 pathology, linking molecular processes, cellular mechanisms, pathological manifestations, and macroscopic brain structure. These findings highlighted the disparate and shared molecular underpinnings of various genetic subgroups and their relationship with brain structural remodeling. With consideration of specific genetic variants involved, this approach can unravel the intricate molecular mechanisms driving the heterogeneous nature of genetic bvFTD. This knowledge might pave the way for precisely tailored strategies in diagnosis and potential treatments.

## Electronic supplementary material

Below is the link to the electronic supplementary material.

**Additional File 1**: **Supplementary Figs. 1–4**. **Supplementary Tables 1–6**.

**Additional File 2**: **Supplementary Table 3**. Significant genes associated with cortical thinning in different genetic forms of bvFTD.

**Additional File 3**: **Supplementary Table 4**. Detailed results of functional enrichments analyses using Metascape.

**Additional File 4**: **Supplementary Table 5**. Overlapped genes between pathology-related genes and identified significant PLS1 genes.

**Additional File 5**: **Supplementary Table 6**. Detailed results of functional enrichments analyses for overlapped genes in Supplementary Table 5.

## Data Availability

The gene expression datasets used are available in the AHBA database (https://human.brain-map.org/), the HPA database (https://www.proteinatlas.org/). The volumetric PET images for synaptic density are available at (https://github.com/netneurolab/hansen_receptors). Other datasets used and/or analyzed during the current study are available from the corresponding author on reasonable request and approval from the Penn Neurodegenerative Data Sharing Committee. Requests may be submitted using a webform request: https://www.pennbindlab.com/data-sharing.

## Abbreviations

bvFTD: Behavioral variant frontotemporal degeneration
AHBA: Allen Human Brain Atlas
INDD: Integrated Neurodegenerative Disease
MMSE: Mini-Mental Status Examination
ALS: Amyotrophic lateral sclerosis
PSP: Progressive supranuclear palsy
PBAC: Philadelphia Brief Assessment of Cognition
MPRAGE: Magnetization-prepared rapid gradient-echo
TR: Repetition time
TE: Echo time
PLS: Partial least squares
PLS1: First PLS component
GO: Gene Ontology
KEGG: Kyoto Encyclopedia of Genes and Genomes
CORUM: Comprehensive Resource of Mammalian protein complexes
BH-FDR: Benjamini-Hochberg false discovery rate
DEGs: Differentially expressed genes
PPI: Protein-protein-interaction
GTEx: Genotype-tissue expression
nTPM: Normalized transcript per million
VIP: Variable importance in projection
GABAergic: Gamma-aminobutyric acid-ergic
PD: Parkinson’s disease
svPPA: Semantic variant primary progressive aphasia
