# Supplementary material for: Disparate and shared transcriptomic signatures associated with cortical atrophy in genetic behavioral variant frontotemporal degeneration
Source: Mol Neurodegener. 2025 Feb 7;20:17. doi: 10.1186/s13024-025-00806-3 (PMC11806866; doi:10.1186/s13024-025-00806-3)
Supplement: Supplementary file 1 — Additional File 1: Supplementary Figs. 1–4. Supplementary Tables 1–6. [file 13024_2025_806_MOESM1_ESM.pdf]

Disparate and shared transcriptomic signatures associated with cortical atrophy in genetic bvFTD

Ting Shen[1], Jacob W. Vogel[2], Vivianna M Van Deerlin[3], EunRan Suh[3], Laynie Dratch[1], Jeffrey S. Phillips[1], Lauren Massimo[1], Edward B. Lee[3], David J. Irwin[1] and Corey T. McMillan[1]

[1] Penn Frontotemporal Degeneration Center, Department of Neurology, Perelman School of Medicine, University of Pennsylvania, Philadelphia, PA, USA

[2] Department of Clinical Sciences Malmö, SciLifeLab, Lund University, Lund, Sweden

[3] Center for Neurodegenerative Disease Research, Department of Pathology and Laboratory Medicine, Perelman School of Medicine, University of Pennsylvania, Philadelphia, PA, USA

- **Supplementary Figures 1-4**
- **Supplementary Table 1-6**

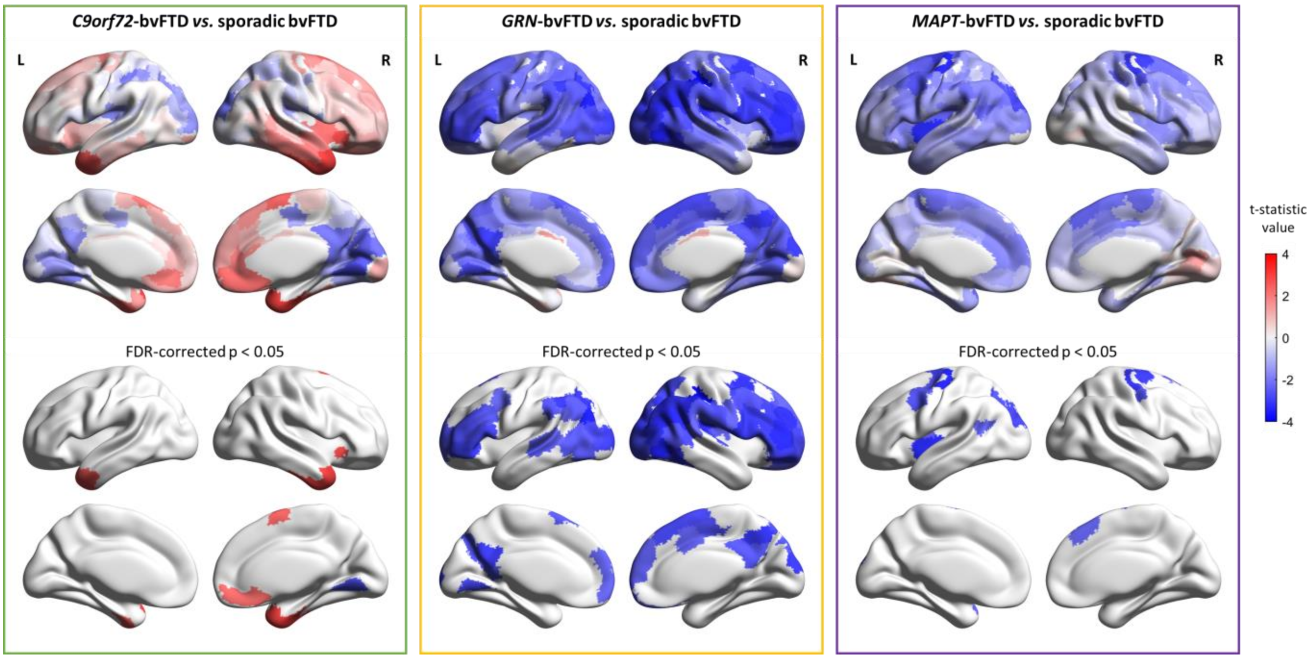

Supplementary Fig. 1 Comparison of cortical thickness between genetic and apparently sporadic bvFTD. For each panel, the brain heatmap on top showing the t-statistic values of all brain regions, and the brain heatmap on bottom showing the regions with significant differences compared to apparently sporadic bvFTD. L, left-hemisphere; R, right-hemisphere.

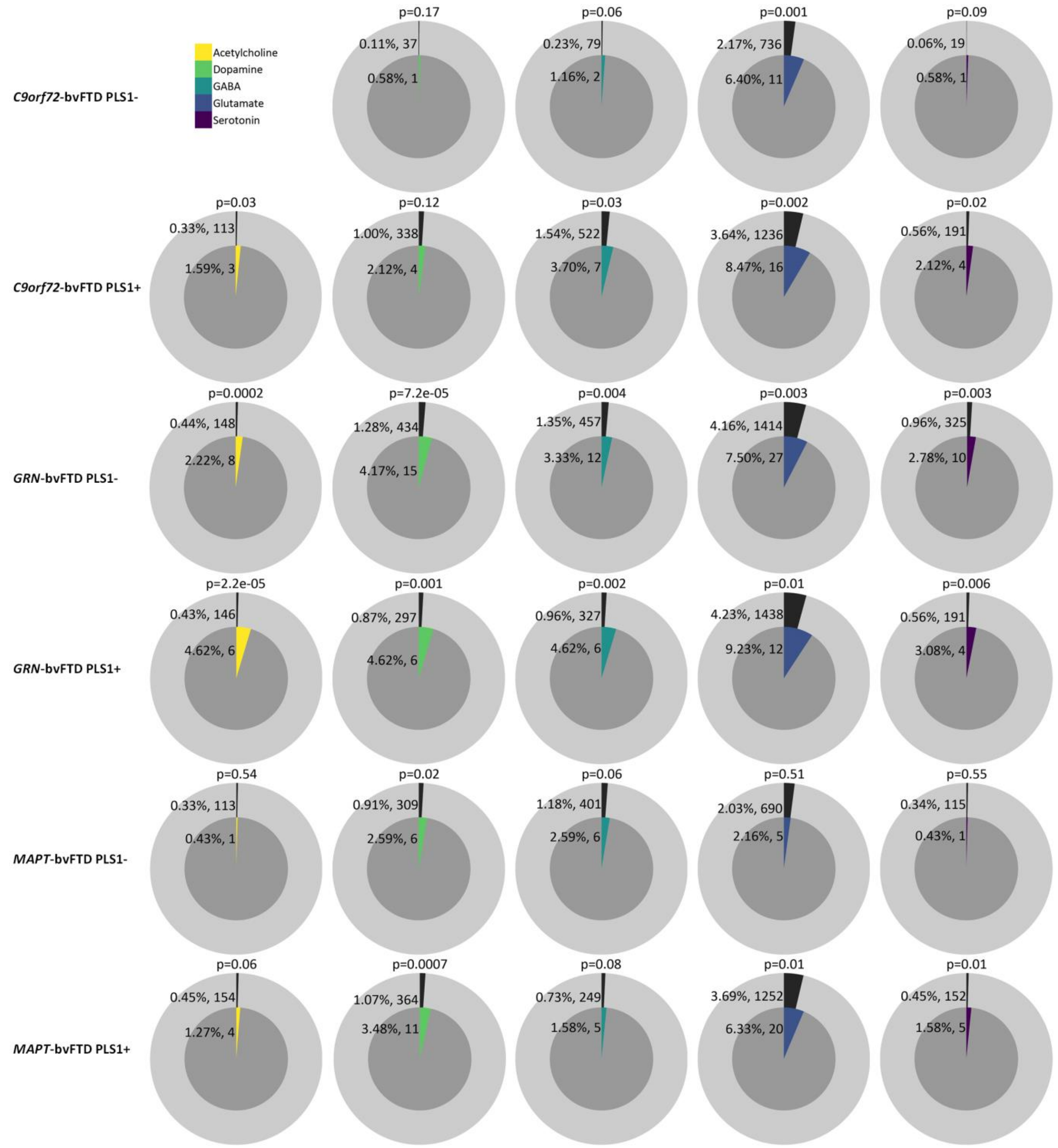

Supplementary Fig. 2 Metascape membership analysis for PLS1-/+ gene lists associated with cortical thickness signatures of each genetic form of bvFTD. Membership search was conducted for terms related to a cholinergic, dopaminergic, GABAergic, glutamatergic, and serotonergic neurotransmitters. The outer ring of each pie represents the number and percentage of genes that are members of selected ontology terms, the inner ring shows the number and percentage of genes in the PLS1-/+ gene lists that are members of selected ontology term. The *p values* at the top of each pie indicates whether the selected term is statistically enriched in the input gene list.

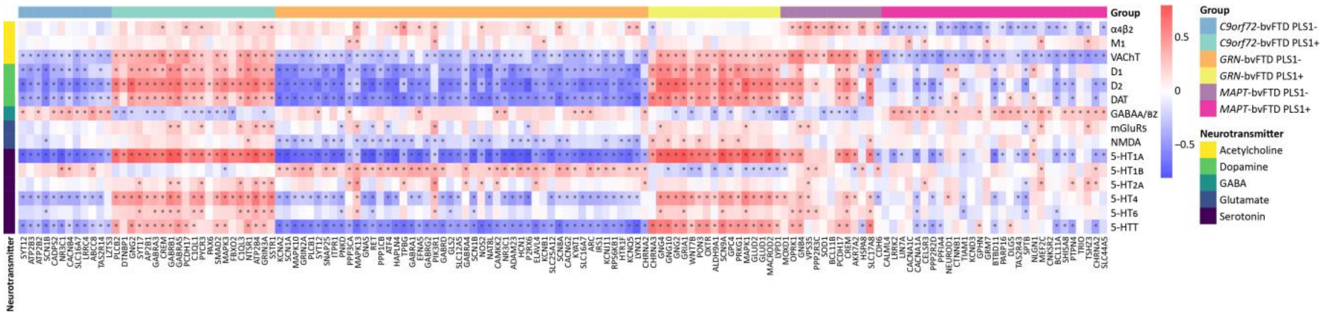

Supplementary Fig. 3 Correlations between the gene expression maps and neurotransmitter receptor/transporter density maps. * indicates that the correlation is significant.

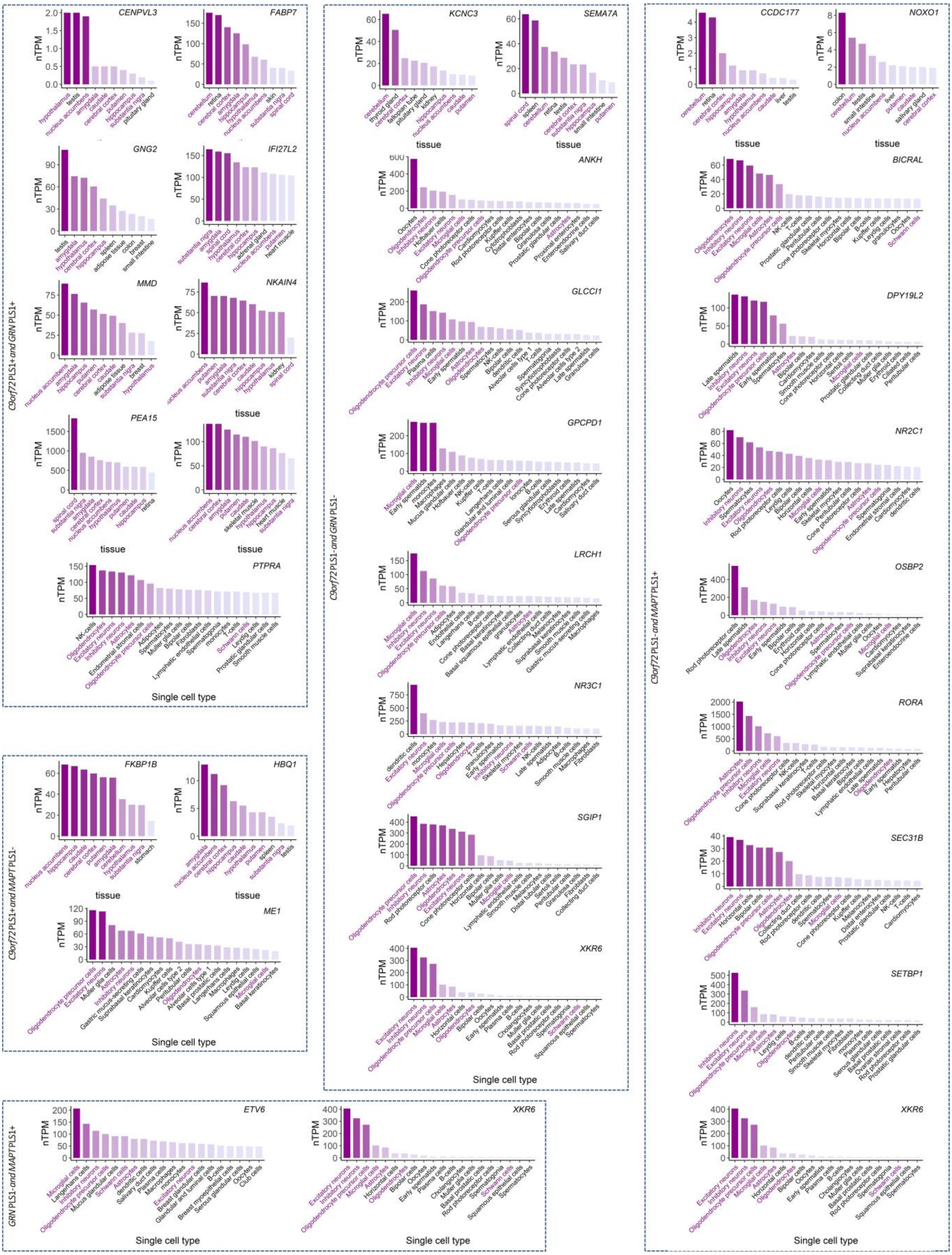

Supplementary Fig. 4 Expression patterns of genes in multiple tissues and cell types. Only genes specifically enriched in either brain tissues or neural cells were shown.

Supplementary Table 1. Profiles of brain donors in AHBA database

**Donor**	**Age at death, years**	**Sex**	**Ethnicity**
H0351.2001	24	Male	Black or African American
H0351.2002	39	Male	Black or African American
H0351.1009	57	Male	White or Caucasian
H0351.1012	31	Male	White or Caucasian
H0351.1015	49	Female	Hispanic
H0351.1016	55	Male	White or Caucasian

Supplementary Table 2. Summary of genetic mutations in individuals with genetic bvFTD

**IDs**	**Gene**	**Mutation Description**
FTDC1-FTDC32	*C9orf72*	GGGGCC repeat expansions (>30 repeats)
FTDC33	*GRN*	*GRN* c.295_308delTGCCCACGGGGCTT, p.C99Pfs*15
FTDC34	*GRN*	*GRN* c.328C>T, p.R110*
FTDC35	*GRN*	*GRN* c.1252C>T, p.R418*
FTDC36	*GRN*	*GRN* c.1179+2T>C
FTDC37	*GRN*	*GRN* c.102delC, p.G35Efs*19
FTDC38	*GRN*	*GRN* c.349+1G>C
FTDC39	*GRN*	*GRN* c.328C>T, p.R110*
FTDC40	*GRN*	*GRN* c.102delC, p.G35Efs*19
FTDC41	*GRN*	*GRN* c.1009C>T, p.Q337*
FTDC42	*GRN*	*GRN* c.299del, p.P100Hfs*156
FTDC43	*GRN*	*GRN* c.814C>A, p.S205*
FTDC44	*MAPT*	*MAPT* c.902C>T, p.P301L
FTDC45	*MAPT*	*MAPT* c.902C>T, p.P301L
FTDC46	*MAPT*	*MAPT* c.915+16C>T
FTDC47	*MAPT*	*MAPT* c.1165G>A, p.G389R
FTDC48	*MAPT*	*MAPT* c.915+16C>T
FTDC49	*MAPT*	*MAPT* c.915T>C, p.S305=
FTDC50	*MAPT*	*MAPT* c.902C>T, p.P301L
FTDC51	*MAPT*	*MAPT* c.915+16C>T
FTDC52	*MAPT*	*MAPT* c.1216C>T, p.R406W
FTDC53	*MAPT*	*MAPT* c.915+16C>T
FTDC54	*MAPT*	*MAPT* c.902C>T, p.P301L
FTDC55	*MAPT*	*MAPT* c.1216C>T, p.R406W
FTDC56	*MAPT*	*MAPT* c.915+16C>T

Supplementary Table 3: Significant genes associated with cortical thinning in different genetic forms of bvFTD. - See Excel sheet

Supplementary Table 4: Detailed results of functional enrichments analyses using Metascape. - See Excel sheet

Supplementary Table 5: Overlapped genes between pathology-related genes and identified significant PLS1 genes. - See Excel sheet

Supplementary Table 6: Detailed results of functional enrichments analyses for overlapped genes in Supplementary Table 5. - See Excel sheet
